# Evaluating treatment response to neoadjuvant chemoradiotherapy in rectal cancer using various MRI-based radiomics models

**DOI:** 10.1186/s12880-021-00560-0

**Published:** 2021-02-16

**Authors:** Zhihui Li, Xiaolu Ma, Fu Shen, Haidi Lu, Yuwei Xia, Jianping Lu

**Affiliations:** 1 Department of Radiology, Changhai Hospital, No.168 Changhai Road, Shanghai, 200433 China; 2 Huiying Medical Technology Co., Ltd, Beijing, China

**Keywords:** Rectal cancer, Neoadjuvant therapy, Radiomics, Magnetic resonance imaging, Machine learning

## Abstract

**Background:**

To validate and compare various MRI-based radiomics models to evaluate treatment response to neoadjuvant chemoradiotherapy (nCRT) of rectal cancer.

**Methods:**

A total of 80 patients with locally advanced rectal cancer (LARC) who underwent surgical resection after nCRT were enrolled retrospectively. Rectal MR images were scanned pre- and post-nCRT. The radiomics features were extracted from T2-weighted images, then reduced separately by least absolute shrinkage and selection operator (LASSO) and principal component analysis (PCA). Four classifiers of Logistic Regression, Random Forest (RF), Decision Tree and K-nearest neighbor (KNN) models were constructed to assess the tumor regression grade (TRG) and pathologic complete response (pCR), respectively. The diagnostic performances of models were determined with leave-one-out cross-validation by generating receiver operating characteristic curves and decision curve analysis.

**Results:**

Three features related to the TRG and 11 features related to the pCR were obtained by LASSO. Top five principal components representing a cumulative contribution of 80% to overall features were selected by PCA. For TRG, the area under the curve (AUC) of RF model was 0.943 for LASSO and 0.930 for PCA, higher than other models (*P* < 0.05 for both). As for pCR, the AUCs of KNN for LASSO and PCA were 0.945 and 0.712, higher than other models (*P* < 0.05 for both). The DCA showed that LASSO algorithm was clinically superior to PCA.

**Conclusion:**

MRI-based radiomics models demonstrated good performance for evaluating the treatment response of LARC after nCRT and LASSO algorithm yielded more clinical benefit.

## Background

Locally advanced rectal cancer (LARC) is routinely managed by neoadjuvant radiotherapy and chemotherapy in combination with total mesorectal resection [1, 2]. Patients’ response to nCRT is of high importance in long-term prognosis and treatment decision making; about 15–27% cases can achieve the pathological complete response (pCR), and are expected to take the treatment measures of observation and waiting [3]. Compared with surgical treatment, the total survival period is not significantly different, and operative complications and mortality are effectively reduced [4, 5]. At present, high-resolution rectal magnetic resonance imaging (MRI) is recommended as an efficient routine imaging technique for evaluating the efficacy of nCRT. However, TRG classification or pCR status determination can only be confirmed by postoperative pathology, and no reliable and accurate evaluation system has been developed for preoperative therapeutic response [6]. Meanwhile, accurate evaluation of the curative effect of preoperative nCRT and early judgment of prognosis would make the treatment more personalized and effective.

Radiomics shows multiple advantages in evaluating therapeutic response over traditional imaging analysis [7–10], thereby providing important details of tissue features [11–19]. Mounting evidence indicates potential benefits for radiomics in assessing therapeutic response in LARC [20–24]. To our knowledge, however, which feature reduction and machine learning model can yield more clinically benefit remains unclear. Therefore, this work aimed to validate and compare different radiomics feature reduction and machine learning models in evaluating the treatment response after nCRT in patients with LARC.

## Methods

### Participants

All methods of the present research were carried out in accordance with the Declaration of Helsinki and were approved by the local Institutional Review Board (Committee on Ethics of Biomedicine, Changhai Hospital, Shanghai, China) Informed consent was waived for this retrospective study. Totally 114 LARC patients examined by rectal MRI and administered surgical resection upon nCRT in our hospital between June 2016 and June 2019 were retrospectively assessed. Inclusion criteria were: (1) histologically confirmed rectal adenocarcinoma with baseline MRI data (≥ cT3 or N +); (2) pre-nCRT MRI within 7 days prior to nCRT and post-nCRT MRI within 7 presurgical days; (3) surgical resection after nCRT completion. Exclusion criteria were: (1) a history of previous malignant tumor or pelvic surgery (n = 3); (2) multiple colorectal cancers (n = 2); (3) poor quality of the images, which could not be used for image segmentation and radiomic feature extraction (n = 11); (4) any other therapy before baseline MR examination (n = 9); (5) interval between nCRT and rectal surgery greater than 12 weeks (n = 9). The trial eventually included 80 cases.

### Imaging acquisition

Rectal MR examination was carried out before and after treatment, respectively, on a 3.0 T MR scanner (including Siemens MAGNETOM Skyra 3.0 T MRI System and GE Discovery MR 750w 3.0 T MRI System) using an abdominal phase array coil. All patients fasted for 4 h before MR examination. Before scanning, intestinal cleaning was performed by enema administration with 20 ml of glycerin. Conventional rectal MR sequences and high-resolution T2W sequences were obtained. Conventional sequences included sagittal T2WI fat suppression sequence, DWI sequence, cross-sectional T1WI and enhanced T1WI. High-resolution T2WI followed an oblique cross-section, with the scanning plane perpendicular to the long axis of the intestinal tract comprising the lesion. The parameters applied for high-resolution T2W sequence, which were used for radiomics models, are presented in Table 1.

**Table 1:** High-resolution T2W sequence acquisition parameters

	TR/TE (m/s)	Matrix	FOV (mm)	Slice thickness/gap (mm)	Bandwidth (Hz) /flip angle (°)	Acquisition times
Siemens	4000/108	320 × 320	180 × 180	3/0	108/150	4 min 10 s
GE	6538/116	320 × 320	180 × 180	3/0	62.5/110	3 min 16 s

### Neoadjuvant chemoradiotherapy treatment

All patients received long-term pelvic radiation therapy with 50.4 Gy in 25–28 fractions plus oral capecitabine (825 mg/m^2^ given twice/day). All patients underwent total mesorectal excision (TME), and were followed up for 8–10 weeks upon treatment completion.

### Pathological evaluation of therapeutic response

Based on the National Comprehensive Cancer Network and American Joint Committee on cancer staging system [25], all pathological stages and tumor regression grades (TRGs) were recorded. TRG was categorized as follows: TRG 0 and TRG 1 as good response group (no residual viable malignant cells, only small cell clusters, or single malignant cells); TRG 2 and TRG 3 as poor response (residual malignant cells with substantial fibrosis, limited/no cancer cell death, or important residual tumor). Pathological complete response (pCR) was reflected by no viable cancer cells in primary tumors or lymph nodes (ypT0N0M0); others constituted the non-pCR group.

### Radiomics feature extraction

**Fig. 1:**
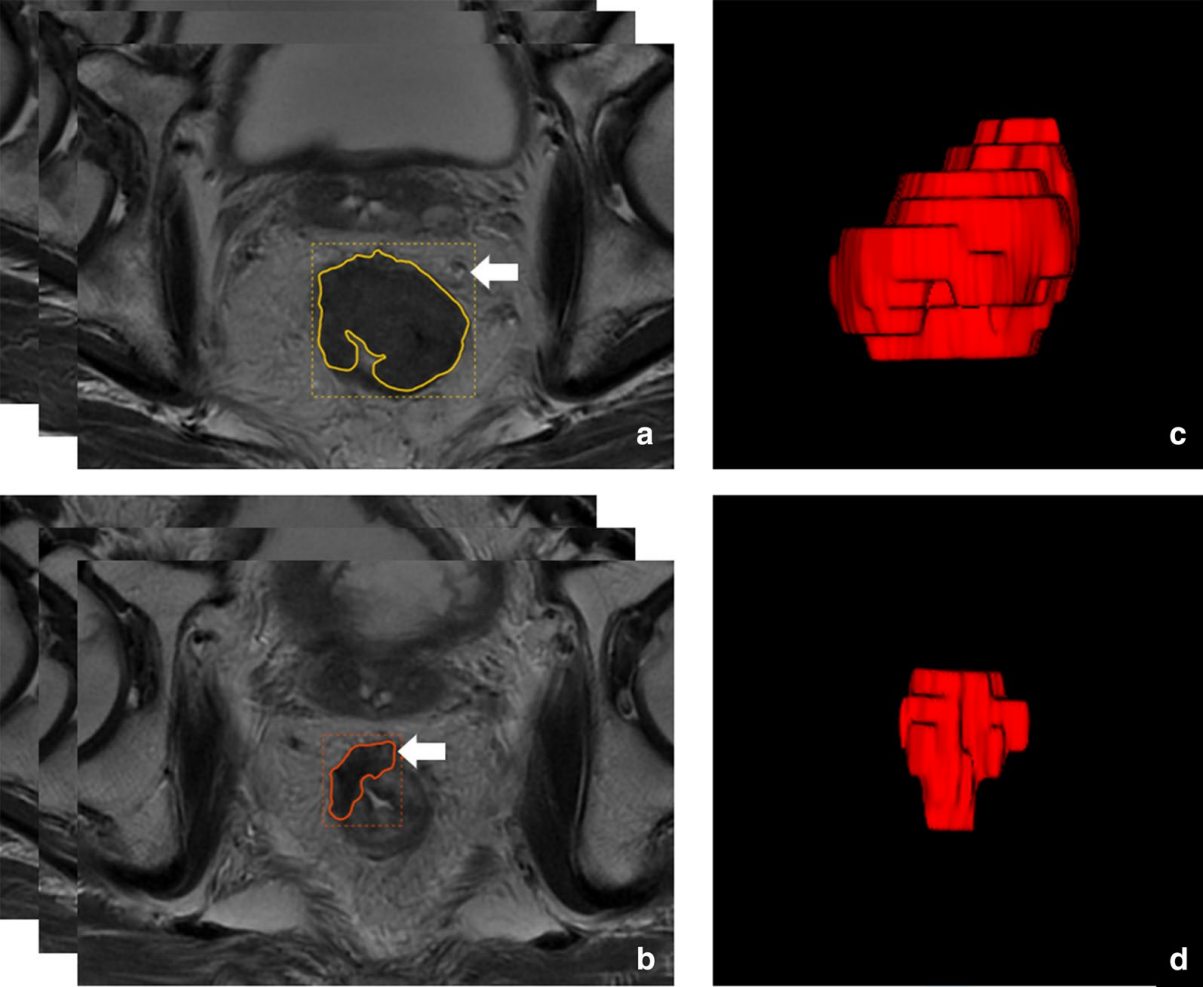
Representative images for lesion contouring. **a**–**d**. Images acquired in a 54-year-old man with LARC, staged as cT3N0. He underwent MRI scanning pre- and post- nCRT. **a**, **b** Delineation of ROIs on oblique-axial T2-weighted MR images pre- and post- nCRT (arrow). **c**, **d** Volume renderings of VOIs pre- and post- nCRT

The original high-resolution T2W DICOM images acquired pre- and post- nCRT were, respectively, imported into the Radcloud radiomics platform (Huiying Medical Technology, Beijing, China). The tumors were manually delineated on each transverse image with the platform. Then, radiomics feature extraction was performed from the volumes of interest pre- and post-nCRT (VOI_pre_ and VOI_post_) (Fig. 1). Each image intensity was normalized to minimize the MRI signal variations.

Using the above platform, radiomics features were obtained based on the “PyRadiomics” package in Python (version 3.0, https://pyradiomics.readthedocs.io/), including four types as follows: (1) first-order statistics (peak and mean values and variance, among others) that quantitate voxel intensity distribution in MR images; (2) shape properties (volume, surface area and spherical value, among others), reflecting the 3D properties of the outlined area’s shape and size; (3) texture properties (gray-level co-occurrence, run length, size zone and neighborhood gray-tone difference matrices), quantifying the selected area’s heterogeneity; (4) higher-order statistics (first-order statistics and texture properties after transformation, i.e., logarithm, exponential, gradient, square, square root, local binary patterns (LBP) and wavelet filters) [26, 27].

### Feature reduction

Two radiologists (H.L. and Z.L., with more than 5 years of experience in rectal MRI) performed image processing of all cases on the platform independently and then reviewed by a senior radiologist (F.S., with 11 years of experience in imaging diagnosis). In addition, one radiologist (Z.L.) repeated the segmentations of 40 cases randomly selected from dataset one week later. The inter- and intraclass correlation coefficient (ICC) was computed for evaluation of the inter-observer reliability and intra-observer reproducibility of features. Features with both inter- and intra-observer ICCs greater than 0.8 were applied for subsequent analysis, which suggested good robustness of features. Then, the variance threshold algorithm (variance threshold selected at 0.8, so that eigenvalues with variance smaller than 0.8 were removed) was applied for further reduction. At last, the least absolute shrinkage and selection operator (LASSO) algorithm and principal component analysis (PCA) were utilized respectively to determine optimal features related to TRG and pCR. The principal components representing a cumulative contribution of 80% to overall features were selected.

### Machine learning and model analysis

Machine learning was performed with the “scikit-learn” package in Python (version 0.23.2, https://scikit-learn.org/stable/), comprising random forest (RF), decision tree (DT), k-nearest neighbor (KNN) and logistic regression (LR) models, leave-one-out cross-validation (LOO-CV) method was adopted for prediction model building based on the optimal features related to TRG classification and pCR, respectively. Details of parameters used in machine learning were shown in Additional file 1: Table S1.

Receiver operator characteristic (ROC) curve generation was performed to assess the performances of various models by calculating areas under the ROC curves (AUCs) in LOO-CV. The Delong test was performed for assessing differences among various classifier models. Decision curve analysis (DCA) was conducted to determine the benefits of radiomics models. *P* < 0.05 indicated statistical significance.

## Results

### Participant characteristics

Totally 80 patients (60 men and 20 women) were assessed. The average age was 56.5 ± 9.5 years. The patient characteristics and pathological outcomes were summarized in Table 2. According to TRG by pathological examination after surgery, 29 patients (36.25%) were classified as good response, including 15 (18.75%) who showed pCR.

**Table 2:** Demographic, pathological and treatment data of the patients

Characteristic	N = 80 (%)
Gender	
Male	60 (75.0%)
Female	20 (25.0%)
Age (year)	
Mean ± SD	56.5 ± 9.5
BMI (kg/m^2^)	
Mean ± SD	24.1 ± 2.6
Tumor location	
Upper	18 (22.5%)
Middle	38 (47.5%)
Lower	24 (30.0%)
ypT stage	
T0	15 (18.75%)
T1	0 (0%)
T2	24 (30.0%)
T3	36 (45.0%)
T4	5 (6.25%)
ypN stage	
N0	47 (58.75%)
N1	15 (18.75%)
N2	18 (22.5%)
CEA^a^	
< 5 ng/ml	29 (36.25%)
≥ 5 ng/ml	51 (63.75%)
CA19-9^a^	
< 37U/ml	44 (55.0%)
≥ 37U/ml	36 (45.0%)
TRG	
TRG 0	15 (18.75%)
TRG 1	14 (17.50%)
TRG 2	32 (40.0%)
TRG 3	19 (23.75%)
pCR	
pCR	15 (18.75%)
Non-pCR	65 (81.25%)

*BMI* body mass index

^a^Preoperative blood samples

### Radiomics features

Totally 1409 radiomics features were obtained from rectal MRI pre- and post- nCRT each, indicating a total of 2818 radiomic features. Totally 2561 features (90.9%) had good robustness (both inter- and intra-observer ICCs ≥ 0.8), and were applied for subsequent analysis.

**Fig. 2:**
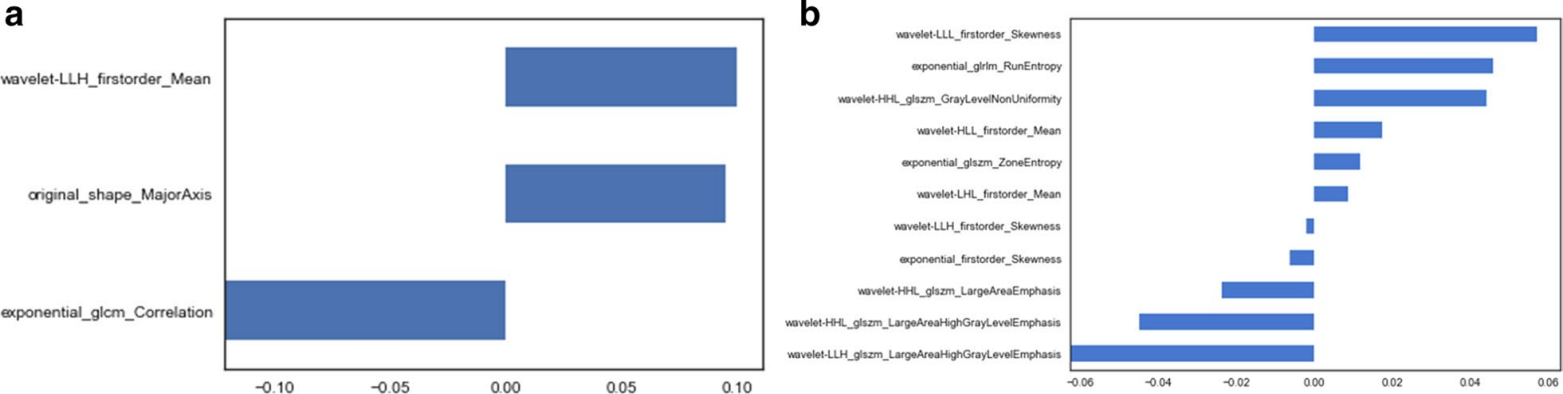
LASSO algorithm for radiomics feature selection. Totally 3 and 11 features for TRG (**a**) and pCR status (**b**) were obtained, respectively

The LASSO algorithm was performed to select vital features. Finally, 3 features related to TRG and 11 features associated with pCR were selected to build the radiomics models (Fig. 2).

**Fig. 3:**
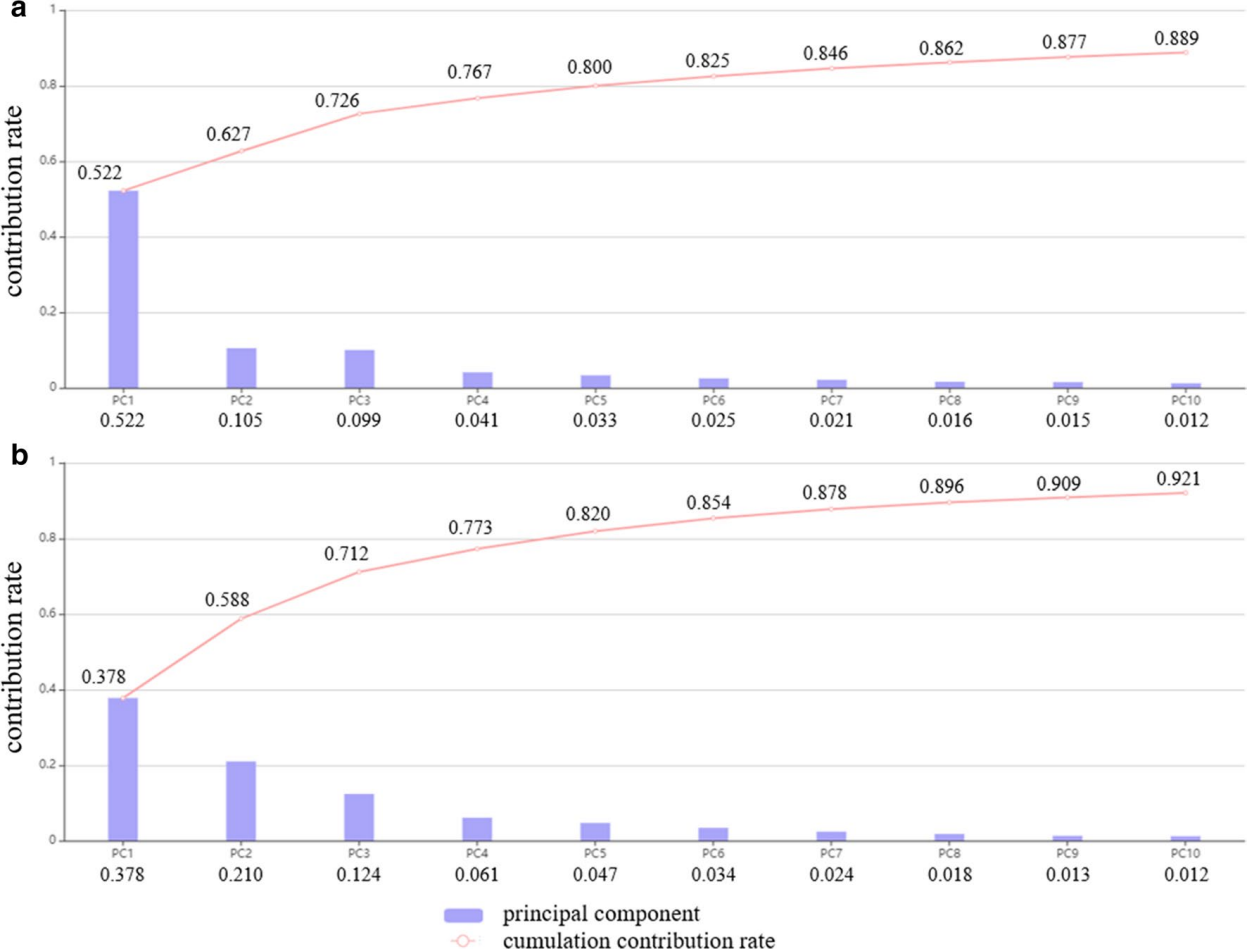
Pareto diagrams of cumulative feature contribution for TRG (**a**) and pCR (**b**). Histograms represent the contribution rates of various principal components. Line graphs depict the cumulative contribution rates of various principal components

Meanwhile, PCA was performed to reduce data dimensionality by identifying new variables, selecting five principal components that can represent a cumulative contribution of 80% to the overall TRG and pCR feature matrix (Fig. 3), respectively.

### Radiomics models of TRG classification

**Fig. 4:**
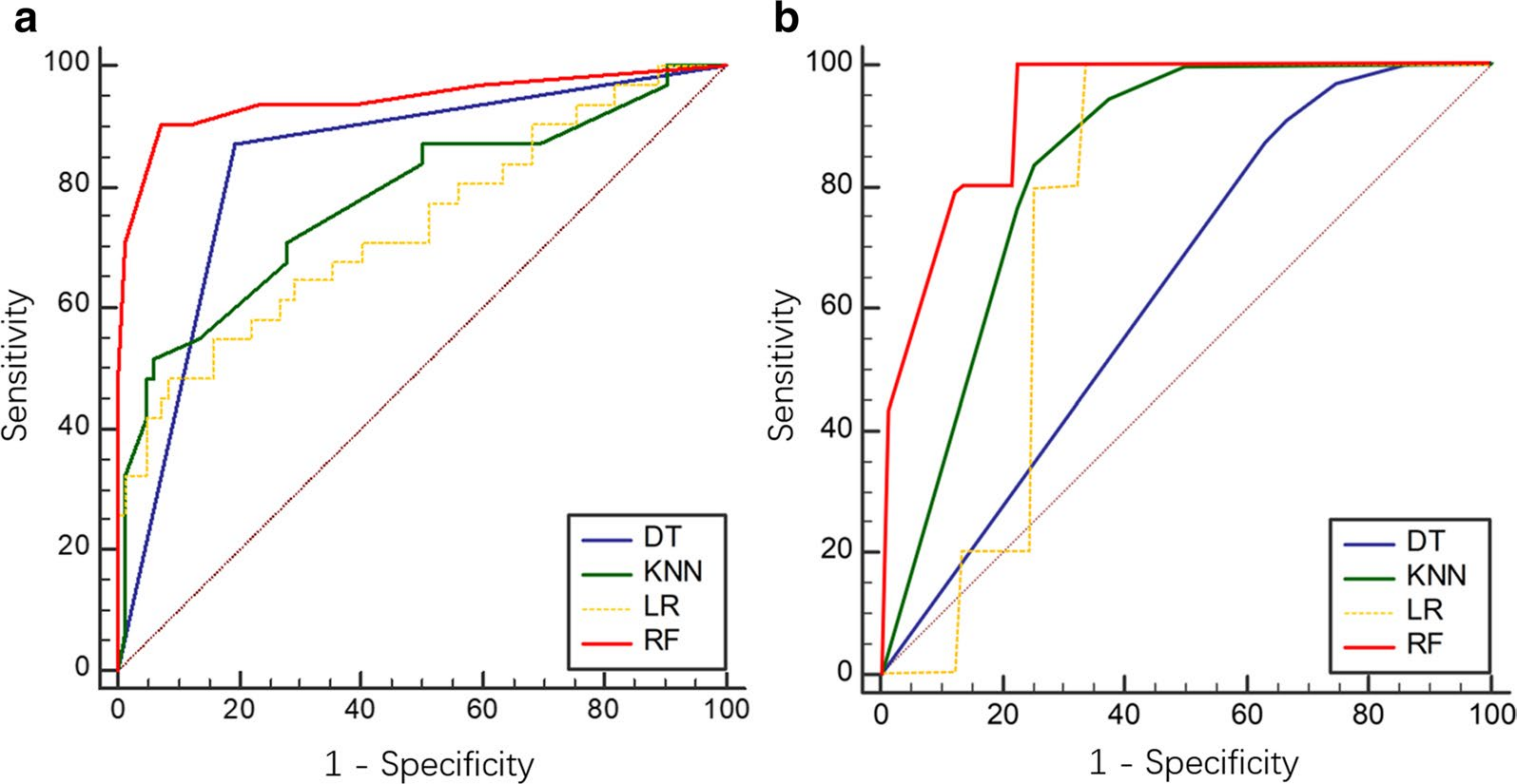
Receiver operator characteristic (ROC) curves of TRG classification models. **a** LASSO algorithm (AUCs were 0.734, 0.943, 0.838 and 0.777 for the LR, RF, DT and KNN models, respectively). **b** PCA (AUCs were 0.761, 0.930, 0.633 and 0.840 for the LR, RF, DT and KNN models, respectively)

In TRG classification, the ROC curves of four models from the LASSO algorithm were shown in Fig. 4a. The RF model had an AUC of 0.943 (95% CI 0.883–0.978), with a sensitivity of 90.3% and a specificity of 92.7%, indicating a better performance compared with the other models. The Delong test showed *P*_RF-LR_ < 0.001, *P*_RF-KNN_ = 0.004 and *P*_RF-DT_ = 0.010, and the other three models were not significantly different (*P* > 0.05). Details contained in the models were shown in Table 3.

**Table 3:** ROC analysis of TRG and pCR classification models

	TRG				pCR			
	AUC	95% CI	Sensitivity (%)	Specificity (%)	AUC	95% CI	Sensitivity (%)	Specificity (%)
LASSO								
LR	0.734	0.643–0.813	48.4	91.5	0.801	0.696–0.882	64.3	84.6
RF	0.943	0.883–0.978	90.3	92.7	0.912	0.827–0.964	78.6	92.3
DT	0.838	0.757–0.901	87.1	80.5	0.870	0.775–0.935	78.6	95.4
KNN	0.777	0.689–0.850	51.6	93.9	0.945	0.870–0.984	85.7	98.5
PCA								
LR	0.761	0.697–0.828	100	0.0	0.597	0.552–0.642	54.0	45.0
RF	0.930	0.849–1.000	90.3	40.0	0.693	0.556–0.830	54.0	35.0
DT	0.633	0.59–0.676	73.3	0.0	0.666	0.570–0.762	40.0	63.3
KNN	0.840	0.788–0.892	100	0.0	0.712	0.557–0.867	78.7	56.7

In the PCA method, the RF model’s AUC was 0.930 (95% CI 0.849–1.000) (Fig. 4b), which was higher than those of other models (Table 3). The Delong test showed *P*_RF-LR_ < 0.001, *P*_RF-KNN_ = 0.002, and *P*_RF-DT_ < 0.001, and the other three models were not significantly different (*P* > 0.05).

### Radiomics models of pCR classification

**Fig. 5:**
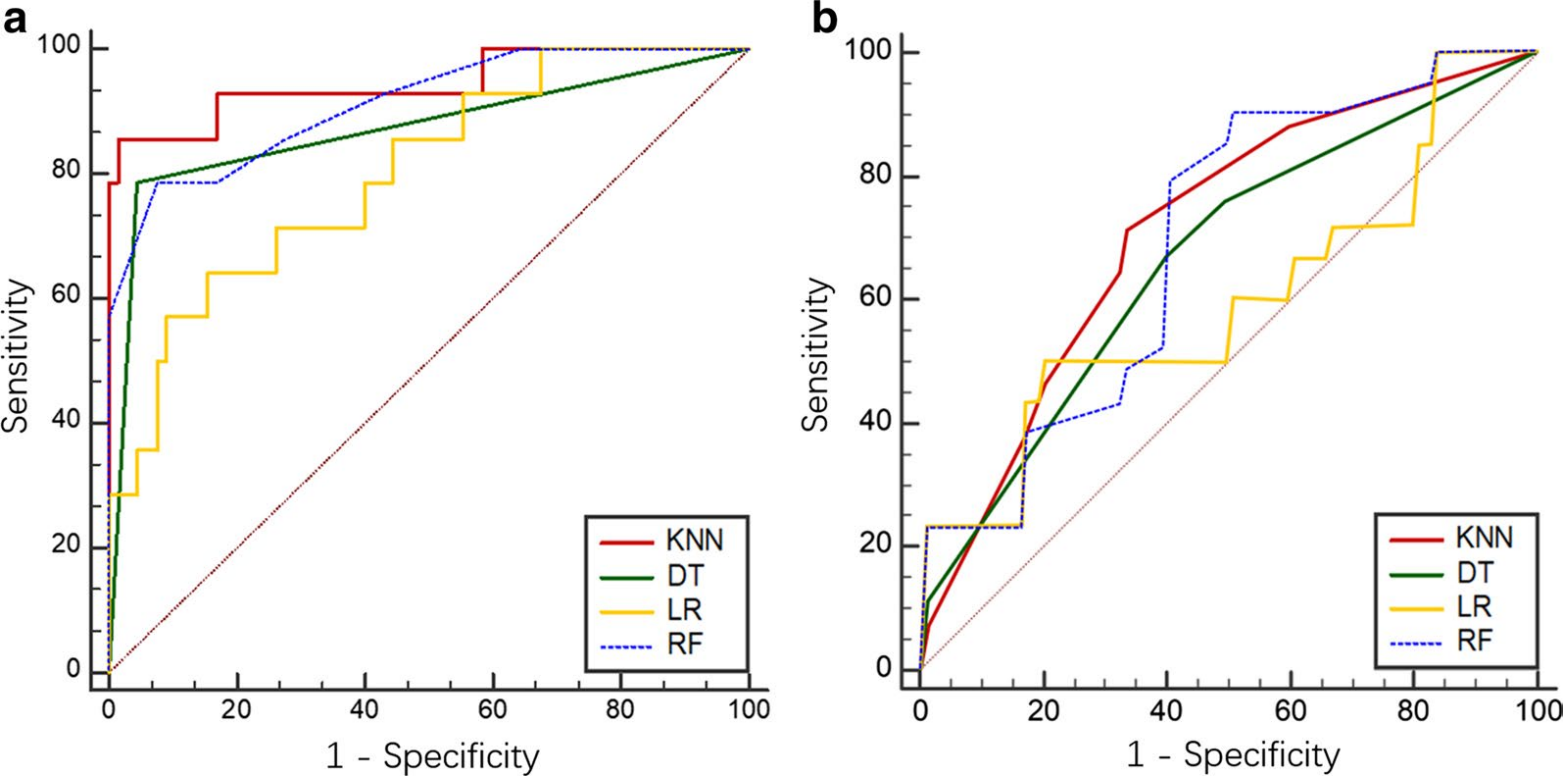
Receiver operator characteristic (ROC) curves for pCR status assessment. **a** LASSO algorithm (AUCs were 0.801, 0.912, 0.870 and 0.945 for the LR, RF, DT and KNN models, respectively). **b** PCA (AUCs were 0.597, 0.693, 0.666 and 0.712 for the LR, RF, DT and KNN models, respectively)

In pCR classification based on the LASSO algorithm, the ROC curves of the four models were shown in Fig. 5a. The KNN demonstrated significantly better performance (AUC = 0.945, 95% CI 0.870–0.984) in the detection of pCR; sensitivity and specificity were 85.7% and 98.5%, respectively. The Delong test yielded *P*_KNN-DT_ = 0.008, *P*_KNN-LR_ = 0.006, and *P*_KNN-RF_ = 0.009, and the other three models were not significantly different (*P* > 0.05). Details were shown in Table 3.

In PCA (Fig. 5b), the comprehensive performance of KNN model was better than those of other classifiers, with an AUC of 0.712 (95% CI 0.557–0.867, Table 3). The Delong test yielded *P*_KNN-LR_ = 0.033, *P*_KNN-RF_ < 0.001, and *P*_KNN-DT_ = 0.048, and the other three models were not significantly different (*P* > 0.05).

### Decision curve analysis

**Fig. 6:**
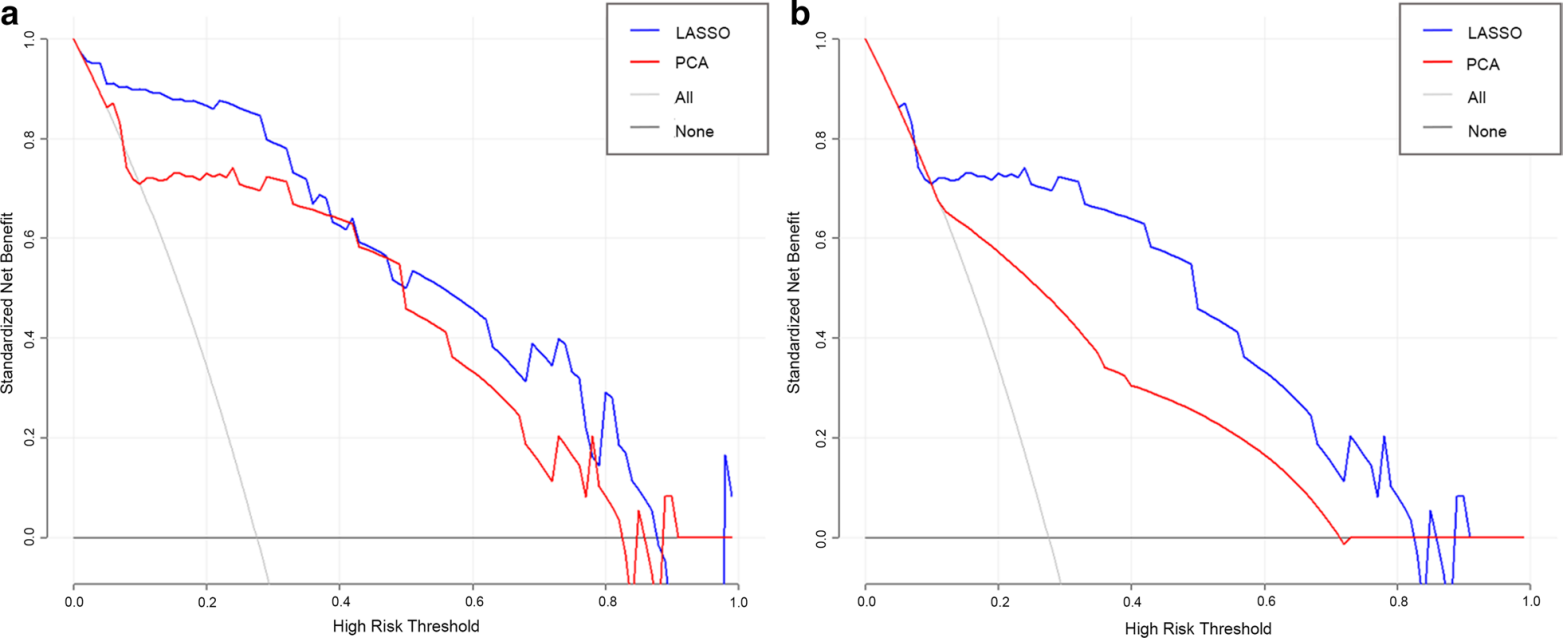
Decision curve analysis (DCA) of the two schemes of reduction. At probability thresholds of 0.0 to 0.9, the RF model based on the LASSO algorithm for TRG classification provided more net benefit than that utilizing PCA (**a**). Except at the probability threshold of 0.4, with comparable benefits from both models (AUCs of 0.943 and 0.930 respectively). Meanwhile, at threshold probabilities of 0.1 to 0.85, the KNN model based on the LASSO algorithm had increased net benefit than the PCA scheme (**b**)

The decision curves demonstrated that for TRG classification, the RF model based on the LASSO algorithm showed a greater advantage compared with the PCA scheme at a threshold probability of 0.0–0.9. However, both models were similar at the probability threshold of 0.4 (Fig. 6a). Meanwhile, DCA showed that at threshold probabilities of pCR ranging from 0.1 to 0.85, the LASSO algorithm added more net benefit than the PCA method (Fig. 6b).

## Discussion

In this study, we obtained radiomics features from rectal high-resolution T2WI images pre- and post- nCRT, respectively. The various machine learning models were shown to constitute an effective non-invasive approach for TRG and pCR assessments in LARC, by both the LASSO algorithm and PCA.

The LASSO algorithm was used for variable filtration and complexity reduction in various models. Finally, 3 features related to TRG and 11 associated with pCR were obtained. Meanwhile, the PCA method was used for feature reduction. The idea behind PCA reduction is to combine the original indexes with a certain correlation into a new set of principal components to replace them. The correlation among multiple variables is investigated; this technique is widely used in applications that need a large number of data processing steps [28]. We performed PCA to reduce the dimensionality of the original features, and the first five principal components which best represented the whole feature matrix were selected for TRG and pCR, respectively. The clinical decision-making curves found that the clinical benefits of the LASSO algorithm were greater than those of the PCA approach in the evaluation of TRG and pCR status.

In recent years, relevant studies have proposed the concept of MR tumor regression classification (mrTRG). Several clinical trials have shown that the imaging grade of tumors is related to the prognosis of patients. Therefore, mrTRG can be used as the main end point with high clinical relevance [6]. The current mrTRG classification system is mainly based on high resolution T2 weighted imaging (T2WI). However, it lacks quantitative evaluation, which leads to low accuracy in predicting the degree of pathological regression [29]. Indeed, the sensitivity and specificity of mrTRG 1/2 for pCR are only 69.9% and 62.2% based on a meta-analysis [30].

Studies have shown that MR-based radiomics models demonstrate good performance in the prediction of treatment response to nCRT in LARC patients [22, 31, 32], indicating that they could help evaluate the post-treatment TRG of rectal cancer. In the current study, individuals with TRG 0 and 1 were classified in the good efficacy group, and TRG 2 and 3 cases were considered as the poor efficacy group. The above analysis indicated that the RF model exhibited a higher predictive performance than the other three models (*P* < 0.05) for TRG classification, with AUCs of 0.943 (LASSO algorithm) and 0.930 (PCA), suggesting good diagnostic efficiency.

**Fig. 7:**
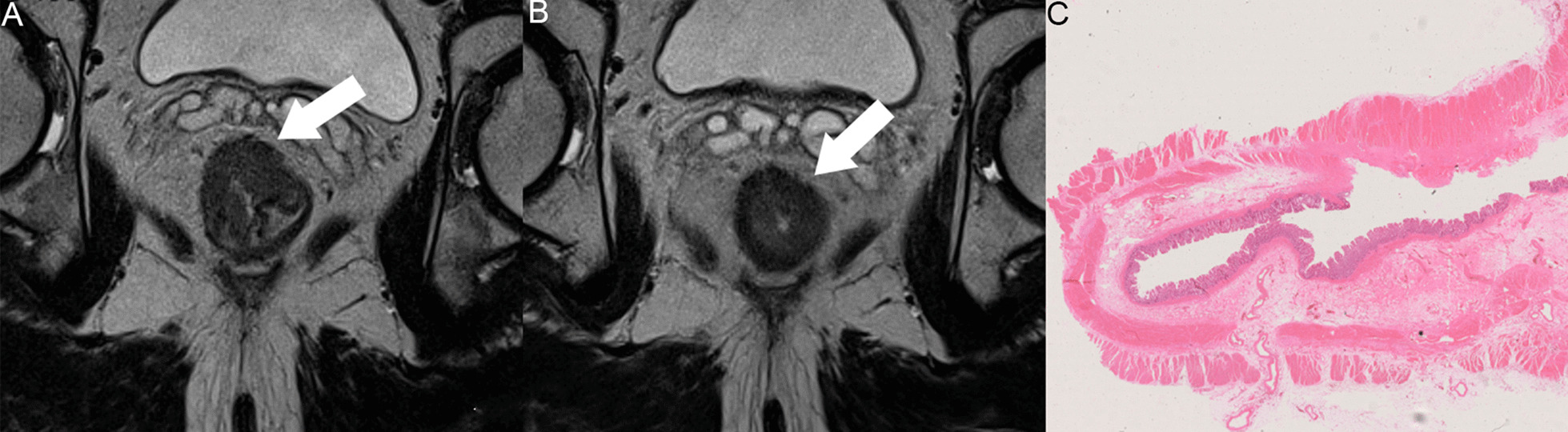
Images acquired in a 64-year-old man with LARC. **a** High resolution T2WI pre-nCRT showed the tumor at the anterior rectal wall (arrow). **b** High resolution T2WI post-nCRT showed obvious tumor regression, with minimal low-signal-intensity residual cells (arrow). The radiomics model suggested a diagnosis of pCR, although a radiologist’s subjective evaluation would call for non-pCR. **c** Postoperative pathological analysis (hematoxylin and eosin, × 1) confirmed this case as pCR

Following nCRT, 15%-27% of LARC cases show no tumor cell survival, which reflects pCR. The long-term prognosis of such individuals is markedly better compared with that of cases with residual tumor cells. The local recurrence rate at 5 years after operation is close to 0%, and the overall survival rate is as high as 95% [4]. Based on high resolution T2WI, sensitivity and specificity of mrTRG 1 for pCR are 32.3% and 93.5%, as suggested by a meta-analysis [30]; this sensitivity was far from satisfactory. However, several studies have shown that MRI-based radiomics models can predict the pCR status effectively [20–24]. Some researchers also combined the pre- and post-nCRT MRI sequence to predict the treatment response using a specific machine learning model, with high predictive value for pCR status evaluation. In our study, different radiomics feature reduction and machine learning models based on T2W images before and after treatment were compared, some of them showed good performance in the evaluation of pCR in patients with LARC (Fig. 7). Among them, the KNN model was better than the other three classifiers (*P* < 0.05) with an AUC of 0.945 (LASSO algorithm), and sensitivity and specificity of 85.7% and 98.5%, respectively. Identifying individuals with elevated odds of pCR preoperatively could help reassess the need for TME, since pCR cases post-resection and the “W&W” group show comparable long-term survival rates.

This study had some limitations. Firstly, VOIs were manually rather than semi-automatically/automatically delineated, making it difficult to avoid the impact of intestinal wall deformation, which is prone to subjective errors; this is not suitable for large-scale data processing [33, 34]. Secondly, this was a retrospective single-center study. The main limitation was the lack of external validation, with relatively few patients and sample distribution was not uniform. Therefore, large multicenter trials are needed to reduce the impact of data bias on model accuracy [35, 36]. Finally, this study did not include relevant clinical influencing factors, such as tumor markers and other molecular biological indicators [37], which deserves further investigation.

## Conclusion

Overall, using high resolution T2WI data before and after neoadjuvant chemoradiotherapy, predictive radiomics models were built based on various machine learning, and demonstrated great performance. Such models can be applied for assessing the treatment response of LARC after nCRT to aid clinicians make appropriate treatment decisions, especially the LASSO algorithm yielded more clinical benefit in feature reduction.

## Supplementary Information

**Additional file 1: Supplemental Table 1**. Details of parameters used in machine learning.

## Data Availability

The datasets used and/or analysed during the current study are available from the corresponding author on reasonable request.

## Abbreviations

nCRT: Neoadjuvant chemoradiotherapy
LARC: Locally advanced rectal cancer
pCR: Pathological complete response
MRI: Magnetic resonance imaging
TRG: Tumor regression grade
LASSO: Least absolute shrinkage and selection operator
PCA: Principal component analysis
LR: Logistic Regression
RF: Random Forest
DT: Decision Tree
KNN: K-nearest neighbor
AUCs: Areas under the curves
TME: Total mesorectal excision
TRGs: Tumor regression grades
ICC: Intraclass correlation coefficient
ROC: Receiver operator characteristic
DCA: Decision curve analysis
mrTRG: MR tumor regression classification
T2WI: T2 weighted imaging
LOO-CV: Leave-one-out cross-validation
