# Supplementary material for: Evaluating treatment response to neoadjuvant chemoradiotherapy in rectal cancer using various MRI-based radiomics models
Source: BMC Med Imaging. 2021 Feb 16;21:30. doi: 10.1186/s12880-021-00560-0 (PMC7885409; doi:10.1186/s12880-021-00560-0)
Supplement: Supplementary file 1 — Additional file 1: Supplemental Table 1. Details of parameters used in machine learning. [file 12880_2021_560_MOESM1_ESM.docx]

**Supplemental Table 1. Details of parameters used in machine learning**

| **Model** | **TRG** | **pCR** |
| --- | --- | --- |
| LR | penalty='l2', solver='liblinear' | penalty='l2', solver='liblinear' |
| DT | criterion='gini' | criterion='gini' |
| RF | n_estimators=15 | n_estimators=15 |
| KNN | n_neighbors=5 | n_neighbors=5 |

RF: random forest, DT: decision tree, KNN: k-nearest neighbor, LR: logistic regression

TRG: tumor regression grade; pCR: pathological complete response.
